# Pancitopenia and refeeding syndrome in anorexia nervosa, regarding a clinical case

**DOI:** 10.1097/MD.0000000000045642

**Published:** 2025-11-14

**Authors:** Xin Zou, Chunxiang Wang, Yuan Xue, Ying Guo

**Affiliations:** a School of Clinical Medicine, Shandong Second Medical University, Affiliated Hospital of Shandong Second Medical University, Weifang, China; b Gaomi People’s Hospital, Gaomi, China; c Department of Endocrinology and Metabolic Diseases, Affiliated Hospital of Shandong Second Medical University, Weifang, China.

**Keywords:** anorexia nervosa, gelatinous bone marrow, hematopoietic function, hypophosphatemia

## Abstract

**Rationale:**

Anorexia nervosa is an eating disorder characterized by persistent energy restriction, distorted body image perception, and intense fear of weight gain. It frequently leads to severe malnutrition and multisystemic impairment. Prolonged energy deficiency disrupts hematopoietic function, resulting in pancytopenia. Refeeding syndrome represents a significant complication during nutritional support for anorexia nervosa patients, with hypophosphatemia potentially causing further hematopoietic damage.

**Patient concerns:**

A 32-year-old female patient with anorexia nervosa was admitted for “pancytopenia and chronic weight loss,” presenting with a body mass index of 11.7 kg/m^2^. Four years prior, workplace stress triggered abnormal eating behaviors, resulting in a rapid weight loss from 45 to 30 kg within 2 months. Over the preceding 2 days, she reported worsening discomfort in her back and precordial region.

**Diagnoses:**

Investigations revealed pancytopenia, although a previous bone marrow aspiration had shown no abnormalities. The patient was diagnosed with anorexia nervosa and pancytopenia secondary to refeeding syndrome.

**Interventions:**

On day 3, phosphate reached a minimum of 0.32 mmol/L (enteral nutrition at 20 kcal/kg/d). A stepwise protocol was initiated, increasing enteral nutrition by 5 kcal/kg every 24 hours, reaching 30 kcal/kg/d by day 5 and maintained thereafter. Concurrent intravenous phosphate supplementation (0.6 mmol/kg/d) was initiated.

**Outcomes:**

At discharge, body weight increased to 31 kg with an elevated reticulocyte count, though the white blood cell count remained low. At 1-month follow-up, hemoglobin rose to 80 g/L and white blood cells to 3.1 × 10^9^/L. However, issues persisted with inadequate protein intake and noncompliance with psychological interventions; the patient refused bone marrow aspiration and outpatient psychological therapy.

**Lessons:**

A stepwise calorie escalation protocol effectively corrects hypophosphatemia in refeeding syndrome. Treatment requires multidisciplinary management and psychological support. Hematopoietic recovery in anorexia nervosa patients with refeeding syndrome necessitates an extended duration. The impact of higher calorie feeding on hematopoiesis warrants further investigation.

## 1. Introduction

Anorexia nervosa (AN) is an eating disorder characterized by deliberate energy restriction, distorted body image perception, and intense fear of weight gain. Prolonged nutritional deprivation causes multisystemic damage, with hematological abnormalities being particularly prominent. Approximately one-third of patients exhibit anemia or neutropenia, while 5% to 10% present with thrombocytopenia. Gelatinous transformation of the bone marrow is recognized as the typical pathological basis for such hematopoietic dysfunction.^[1]^ Nutritional support is a critical intervention for AN recovery, yet inappropriate refeeding protocols may precipitate refeeding syndrome (RFS).

Current clinical debate regarding refeeding protocols for AN patients centers on initial calorie selection. While traditional low-calorie regimens remain widely employed, multiple studies confirm their limited efficacy in promoting weight restoration and improving hematopoietic function.^[2]^ Higher-calorie initiation protocols may offer greater advantages for weight gain, yet their adoption remains limited due to concerns over refeeding syndrome implications.^[3]^ The relative advantages of low- versus high-calorie feeding protocols for AN patients remain incompletely defined.

The American Society for Parenteral and Enteral Nutrition (ASPEN) and the UK National Institute for Health and Care Excellence (NICE) have established clear RFS risk assessment criteria, providing key guidance for clinical decision-making. The ASPEN criteria classify risk into moderate and major categories: Moderate risk requires fulfillment of 2 criteria (body mass index [BMI] 16–18.5 kg/m^2^, ≥5% weight loss within 1 month, ≥5–6 days without oral intake or extremely low caloric intake, single supplementation of baseline electrolyte lower limit, moderate subcutaneous fat/muscle loss, or concomitant moderate illness); major risk requires fulfillment of 1 criterion: BMI < 16 kg/m^2^, weight loss ≥7.5% within 3 months or >10% within 6 months, >7 days without oral intake or extremely low caloric intake, multiple corrections of moderately low baseline electrolytes, severe subcutaneous fat/muscle loss, or coexisting severe disease.^[4]^ NICE criteria explicitly state: meeting any 1 of BMI < 16 kg/m^2^, malnutrition exceeding 10 days, weight loss ≥ 15% over 3 to 6 months, or pre-feeding electrolyte/vitamin deficiency; or meeting any 2 of BMI < 18.5 kg/m^2^, unintended weight loss > 10% over 3 to 6 months, minimal food intake within 5 days, and history of alcohol/ drug abuse history.^[5]^ Both guidelines further emphasize that high-risk patients require intravenous thiamin (200–300 mg/d) prior to or concurrent with nutritional support. This supplementation replenishes a key coenzyme in carbohydrate metabolism, preventing complications such as Wernicke encephalopathy caused by thiamin depletion.

Against this backdrop, this paper reports a case of AN complicated by pancytopenia and RFS. Utilizing specific numerical thresholds from ASPEN/NICE criteria, the patient was classified as extremely high-risk. The study analyses the efficacy of a stepwise calorie escalation protocol combined with intravenous thiamin prophylaxis, while investigating the impact of abnormal bone marrow adipose tissue on hematopoietic recovery. This provides clinical guidance for the safe implementation and optimization of nutritional support in AN patients.

## 2. Patient information and clinical findings

This study was approved by the Ethics Committee of the Affiliated Hospital of Shandong Second Medical University (approval no. SDSMU-2025-qt-55). The patient provided written informed consent for the use of her clinical data in this case report. The study complies with the ethical principles of the Declaration of Helsinki.

**Figure 1.**
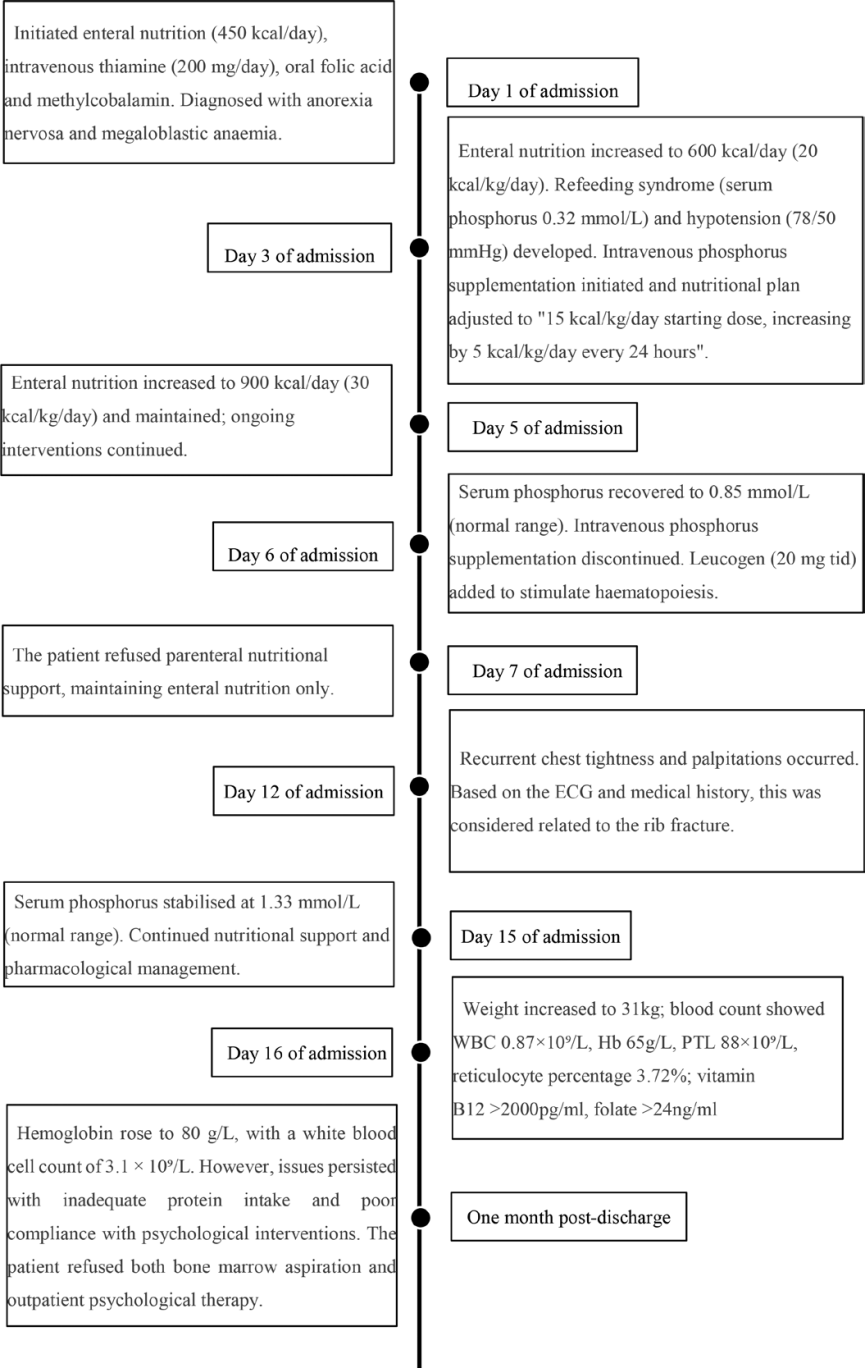
Timeline of events during hospitalization and follow-up.

**Figure 2.**
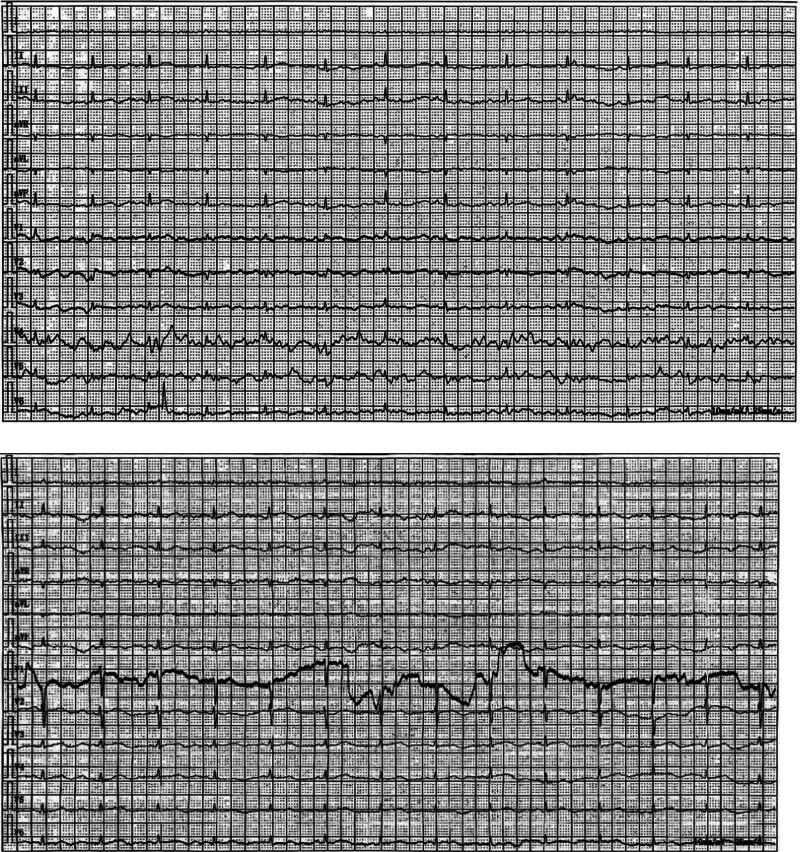
Electrocardiogram at admission (day 1 and day 12).

A 32-year-old female was admitted to our ward presenting with pancytopenia and cachexia. Following workplace bullying 4 years prior, she developed persistent depression, chronic insomnia, and anxiety. Her dietary pattern was monotonous, consisting primarily of eggs, vegetables, and noodles, with daily caloric intake of approximately 500 to 800 kcal (significantly below the average adult female requirement of 1800–2200 kcal). Over 2 months, her weight plummeted from 45 to 30 kg. She had previously consulted multiple external hospitals for anemia and fatigue. In August 2020, a bone marrow biopsy was performed due to pancytopenia, revealing no evidence of malignancy. A follow-up bone marrow examination in 2021 again showed no definitive signs of malignancy. In 2023, prolonged bed rest due to a fracture significantly reduced daily activity, exacerbating muscle wasting and nutritional deficiency. On admission, physical examination revealed: temperature 36.2°C, pulse rate 57 beats per minute, respiratory rate 19 breaths per minute, blood pressure 80/58 mm Hg, height 160 cm, markedly emaciated physique (as shown in Fig. 1), BMI 11.7 kg/m^2^, alert and oriented, anemic appearance, pallor of skin and mucous membranes throughout, scattered petechiae in the oral cavity, multiple decubitus ulcers in the sacrococcygeal region, pharyngeal hyperemia, slightly diminished breath sounds in both lungs, soft abdomen with a barquine shape, no edema in both lower limbs. Admission laboratory results (as per Table 1). Electrocardiogram demonstrated sinus rhythm with low-voltage QRS complexes in limb and left chest leads (as per Fig. 2). Echocardiography revealed no evidence of myocardial damage, showing only mild tricuspid regurgitation. Based on the patient’s symptoms, medical history, laboratory findings, and consultation of DSM-5 guidelines,^[6]^ we considered the diagnosis of anorexia nervosa. Regarding the patient’s pancytopenia, we contemplated bone marrow aspiration for definitive diagnosis. However, the patient categorically refused this procedure due to previous normal results. Consequently, we pursued differential diagnoses (as per Table 2), ultimately considering megaloblastic anemia the most probable diagnosis. Thus, the patient’s provisional diagnosis is anorexia nervosa and megaloblastic anemia.

**Table 1:** Patient admission laboratory results.

Specific examination items	Result	Reference range
White blood cells	0.99 × 10^9^/L	3.5–9.5 × 10^9^/L
Hemoglobin	70 g/L	115–150 g/L
Platelets	94 × 10^9^/L	125–350 × 10^9^/L
Absolute reticulocyte count	0.05 × 10^12^/L	0.024–0.084 × 10^12^/L
Percentage of reticulocytes	2.43%	0.5%–1.5%
Folate	2.8 ng/mL	5.21–19.2 ng/mL
25-Hydroxyvitamin D	12.77 ng/mL	>20 ng/mL
PT	14.3 s	9.4–12.5 s
d-dimer	1.118 mg/L	0–0.5 mg/L
Alanine aminotransferase	18.9 U/L	7–40 U/L
Albumin	34.5 g/L	40–55 g/L
Creatinine	18.2 μmol/L	41–73 μmol/L
Calcium	2.04 mmol/L	2.11–2.52 mmol/L
Potassium	3.67 mmol/L	3.5–5.3 mmol/L
BNP	189.57 pg/mL	0–100 pg/mL
Serum ferritin	151.00 ng/mL	7–227 ng/mL
LDH	230.3 U/L	120–250 U/L
IBIL	5.47 μmol/L	0–17 μmol/L

BNP = B-type natriuretic peptide, IBIL = indirect bilirubin, LDH = lactate dehydrogenase, PT = prothrombin time.

**Table 2:** Differential diagnosis of pancytopenia.

Differential diagnostic dimensions	Target disease	Evidence of nutritional deficiency	Evidence of bone marrow suppression	Key exclusion criteria	Conclusion
Insufficient hematopoietic raw materials	Megaloblastic anemia (folate deficiency type)	Supporting: admission folate 2.8 ng/mL (↓), long-term vegetarian diet, reticulocyte count increased to 3.72% (↑) following folate supplementation	Contrary evidence: previous bone marrow examination showed no abnormalities	Vitamin B_12_ > 2000 pg/mL (normal), ruling out vitamin B_12_-deficient megaloblastic anemia	Confirmed diagnosis (primary cause)
Bone marrow hypoplasia	Gelatinous transformation of bone marrow (AN-associated)	Supporting: BMI 11.7 kg/m^2^ (severe malnutrition), albumin 34.5 g/L (↓)	Supporting: 4-year pancytopenia, slow hematopoietic recovery (discharge WBC 0.87 × 10^9^/L)	Patient declined repeat bone marrow examination; previous bone marrow biopsy showed no typical fatty degeneration	Highly suspected
Myeloid failure	Aplastic anaemia	Not supported: no other hematopoietic substrate deficiency; improvement in parameters following folic acid supplementation	Not supported: reticulocyte count 2.43% (↑), bone marrow biopsy showed no evidence of reduced proliferation	No bone marrow features of hematopoietic failure; antianemic therapy effective	Excluded
Excessive red cell destruction	Hemolytic anemia	Not supported: no evidence of shortened erythrocyte lifespan due to substrate deficiency	Not supported: normal indirect bilirubin and LDH levels, absence of hemolysis signs	Elevated reticulocyte count reflects compensatory hematopoiesis, not excessive destruction	Excluded
Iron metabolism disorder	Chronic disease anemia	Not supported: normal ferritin, no iron metabolism abnormalities	Not supported: no history of chronic inflammation/neoplasms	Macrocytic anemia (MCV 102.4 fL), inconsistent with the “microcytic” nature of the condition	Excluded

AN = anorexia nervosa.

## 3. Diagnostic assessment and therapeutic interventions

Day 1 of hospitalization: Initiated enteral nutrition with intact protein formula (Huiyuan [China Resources Shuanghe], 450 mL/d [450 kcal/d]), oral folic acid 10 mg times daily (tid), methylcobalamin 0.5 mg tid, intravenous thiamin 200 mg/d to prevent RFS; On day 3, when enteral nutrition was increased to 20 kcal/kg/d (600 kcal/d), typical RFS developed with serum phosphorus decreasing to 0.32 mmol/L and hypotension (78/50 mm Hg). Following ASPEN guidelines, a stepwise protocol was initiated with 5 kcal/kg/d increments every 24 hours.^[4]^ On day 5, reached and maintained 30 kcal/kg/d (900 kcal/d) with concurrent intravenous phosphorus supplementation at 0.6 mmol/kg/d; serum phosphorus recovered to 0.85 mmol/L by day 6. Leucogen 20 mg 3 tid was added [generic name: Leucogen tablets; active ingredient: Leucogen; chemical name: 2-(α-phenyl-α-ethoxycarbonylamino)thiazolidine-4-carboxylic acid]. Its mechanism of action involves converting into active thiol compounds in the alkaline environment of the duodenum, which enhances the proliferation and differentiation of hematopoietic stem cells in the bone marrow and promotes the maturation of hematopoietic cells. This medication was used for the patient’s malnutrition-related pancytopenia, as its ability to boost hematopoietic stem cell activity targets her abnormal bone marrow microenvironment. However, the bone marrow hematopoietic stimulation effect has not yet become apparent after treatment, which is considered related to the short medication duration and persistent microenvironment impairment from long-term malnutrition. Electrolyte monitoring (focusing on serum phosphorus) was conducted 3 times during days 5 to 7 of hospitalization: once daily on day 5 (after nutritional dose increased to 30 kcal/kg d), day 6 (following intravenous phosphorus supplementation), and day 7 (routine monitoring). No additional ECG monitoring was performed (no abnormal symptoms such as chest tightness or palpitations). On day 7, the patient declined all recommendations for parenteral nutritional support, accepting only enteral nutrition. From day 12, the patient experienced recurrent chest tightness and palpitations. An ECG was performed (Fig. 2), showing sinus rhythm, considered related to the rib fractures. On day 15, electrolyte results indicated serum phosphorus stabilized at 1.33 mmol/L. Relevant electrolyte data for days 1 to 6 are presented in Table 3.The patient’s clinical course, including caloric intake, electrolyte changes, key interventions, and outcomes, is visually summarized in Figure 1 (timeline of events).

**Table 3:** Electrolyte data for days 1 to 6.

Monitoring period	Serum phosphorus (mmol/L) (normal range: 0.81–1.45)	Serum calcium (mmol/L) (normal range: 2.11–2.52)	Serum potassium (mmol/L) (normal range: 3.5–5.3)	Corresponding therapeutic measures
Day 1 of admission	0.72 (↓)	2.04 (↓)	3.67 (normal)	Initiated enteral nutrition 450 kcal/d + intravenous thiamine supplementation 200 mg/d
Day 2 of admission	0.70 (↓)	2.02 (↓)	3.60 (normal)	Maintained enteral nutrition at 450 kcal/d; no adjustment to regimen
Day 3 of admission	0.32 (↓↓, RFS episode)	1.98 (↓)	3.55 (lower limit of normal)	Enteral nutrition increased to 600 kcal/d; initiated intravenous phosphorus supplementation (0.6 mmol/kg/d)
Day 4 of hospitalization	0.55 (↓)	2.00 (↓)	3.62 (normal)	Continued intravenous phosphorus supplementation; nutritional regimen adjusted to 750 kcal/d
Day 5 of admission	0.78 (approaching normal)	2.05 (↓)	3.70 (normal)	Enteral nutrition increased to 900 kcal/d, continued intravenous phosphorus supplementation
Day 6 of admission	0.85 (normal)	2.08 (approaching normal)	3.75 (normal)	Nutritional regimen maintained at 900 kcal/d

BMI = body mass index.

## 4. Outcomes

At discharge (day 16), weight increased to 31 kg. Blood count showed: WBC 0.87 × 10^9^/L, Hb 65 g/L, platelet 88 × 10^9^/L, reticulocyte percentage 3.72%. Vitamin B12 > 2000 pg/mL and folate > 24 ng/mL in anemia panel. Based on these findings, the following follow-up plan was established: one short-term outpatient visit at 1 month and 2 months post-discharge, followed by long-term follow-up every 3 months commencing from the 3rd month, continuing for 6 to 12 months.

The nutritional support program shall commence at the current tolerated level of 900 kcal/d (30 kcal/kg/d), with gradual weekly increases of 100 to 150 kcal. with the objective of reaching 1200 to 1500 kcal/d within 1 to 2 months. Once stable monthly weight gain of 0.5 to 1 kg is achieved, transition to the recommended adult female energy intake of 1800 to 2000 kcal/d, ultimately raising BMI to above 18.5 kg/m^2^ (exiting severe malnutrition). Protein intake should be adjusted concurrently, commencing at 40 g daily and progressively increasing to 1.0–1.2 g/kg/d (calculated from a discharge weight of 31 kg, with an initial target of 31–37 g daily, subsequently rising to ≥50 g daily as weight increases). This ensures adequate high-quality protein for bodily repair and restoration of hematopoietic function. Complete blood counts at each follow-up visit, focusing on: Gradual restoration of white blood cell count to ≥4.0 × 10^9^/L; Hemoglobin rising to ≥90 g/L within 2 months and ≥110 g/L within 6 months; Maintaining reticulocyte percentage within the normal range of 0.5% to 1.5% maintaining the reticulocyte percentage within the normal range of 0.5% to 1.5%, monitoring anemia-related parameters and electrolytes (particularly maintaining serum phosphorus at 0.81–1.45 mmol/L), and promptly assessing nutritional improvement and hematopoietic recovery. Simultaneously, clearly inform patients and families of potential risks: long-term malnutrition reduces bone density, increasing the risk of aggravated or new rib fractures, necessitating avoidance of strenuous activity; a history of hypotension means inadequate energy intake may trigger circulatory instability, requiring regular blood pressure monitoring; failure to follow the stepwise nutritional escalation protocol may re-induce refeeding syndrome. Poor adherence to psychological interventions elevates the risk of anorexia nervosa recurrence. Insufficient protein intake coupled with refusal of bone marrow aspiration delays hematopoietic recovery, increasing susceptibility to infection and worsening anemia. At 1-month follow-up, hemoglobin rose to 80 g/L and white blood cells to 3.1 × 10^9^/L. However, protein intake remained inadequate (40 g daily) and psychological intervention compliance persisted as an issue. The patient continued to refuse bone marrow aspiration and outpatient psychological therapy.

## 5. Discussion

Anorexia nervosa is a mental disorder characterized by self-induced weight loss through restrictive eating behaviors and/or compensatory behaviors. Altered blood cell counts are common in patients with anorexia nervosa; studies indicate that nearly one-third of patients exhibit anemia or neutropenia, while 5% to 10% present with thrombocytopenia. Gelatinous transformation of the bone marrow has been established as a characteristic feature of hematological alterations in anorexia nervosa patients. However, no direct evidence links changes in blood cell counts to this bone marrow transformation.^[7]^ Abnormal blood cell counts in this patient do not necessarily indicate bone marrow pathology.

A review study indicates partial divergence between high-energy and low-energy feeding regimens in anorexia nervosa patients. Regarding refeeding syndrome incidence, no significant difference was observed between low-calorie and high-calorie groups; indeed, the low-calorie group exhibited a higher probability of onset. However, this does not establish superiority of high-calorie regimens in preventing refeeding syndrome. Regarding hypophosphatemia, its incidence was found to correlate not with caloric intake but with the severity of pre-hospitalization malnutrition. In terms of weight gain, the high-calorie group demonstrated a significant advantage over the low-calorie group, achieving weight gain rates nearly double those of the low-calorie group. Weight gain is intrinsically linked to overall patient recovery, encompassing cognitive function and psychological improvement. Regarding hospitalization duration, the high-energy group demonstrated a substantial advantage.^[8]^

Bone marrow adipose tissue (BMAT), the adipose tissue within the bone marrow cavity, constitutes a vital component of the bone marrow microenvironment. It plays a crucial role in the recovery of hematopoietic function during the refeeding phase following prolonged starvation in patients with AN.^[9]^ Clinical research evidence indicates that under steady-state conditions, body weight negatively correlates with BMAT. Given that most AN patients experience prolonged malnutrition, their BMAT levels are significantly higher than those in normal-weight individuals. This abnormal elevation of BMAT occupies the survival space of hematopoietic stem cells (HSCs) within the bone marrow, disrupts the signaling interactions between HSCs and supporting cells such as osteoblasts and endothelial cells, and simultaneously suppresses the bone marrow’s capacity for acute hematopoietic reconstitution. During periods of subacute weight fluctuation (such as during refeeding therapy), BMAT levels follow a pattern consistent with subcutaneous and visceral fat: they increase with weight gain and decrease with weight loss. This implies that in the early stages of refeeding for this patient, despite weight recovery, BMAT levels may continue to rise with weight gain rather than immediately returning to normal. This persistent compression of the hematopoietic microenvironment would delay HSC proliferation and differentiation,^[10]^ potentially explaining the patient’s suboptimal hematopoietic recovery posttreatment.

It should be particularly noted that, according to the patient’s cardiac echocardiography results, the elevated B-type natriuretic peptide (BNP) levels observed may not be attributable to myocardial damage. This could be related to metabolic abnormalities induced by extreme malnutrition. Based on current literature reports, considering the patient’s baseline condition, her long-term daily caloric intake was only 500 to 800 kcal (far below the 1800–2200 kcal requirement for adult females), with a BMI as low as 11.7 kg/m^2^, representing a classic cachectic state. As a peptide involved in energy metabolism regulation, BNP initiates a dual compensatory mechanism during severe energy deficiency. On one hand, it activates receptors on adipocyte surfaces to promote lipolysis, converting stored fat into free fatty acids for energy to alleviate systemic energy crisis. On the other hand, it indirectly reduces energy expenditure by suppressing ghrelin secretion in the hypothalamus and enhancing satiety. Whilst this adaptive response constitutes the body’s self-protective mechanism against malnutrition, sustained compensatory secretion leads to BNP accumulation in the bloodstream, manifesting as elevated detection values.^[11]^

This patient experienced workplace bullying 4 years prior, disrupting psychological equilibrium and triggering persistent depression, chronic insomnia, anxiety, and other emotional disturbances. During hospitalization, depressive mood persisted; the patient adopted a pessimistic outlook, refusing to cooperate with treatment protocols and rejecting parenteral nutrition support. This impeded comprehensive nutritional intervention. Although physiological issues like hypophosphatemia were corrected via a stepwise calorie escalation protocol, subsequent recovery remained suboptimal. This suggests that when treating anorexia nervosa patients with additional complications, active efforts to address psychological trauma and improve emotional state are equally crucial.

In most cases of cytopenia associated with anorexia nervosa, blood cell counts typically improve with refeeding. However, severe complications of anorexia nervosa, the severity of mental illness, and patient noncompliance can all impact refeeding outcomes and delay cellular recovery.^[12]^ The patient initially demonstrated poor treatment compliance. During intravenous fluid administration, transient chest tightness occurred, clearly related to rib fractures. Subsequently, the patient developed fear, subjectively believing intravenous fluids exacerbated physical discomfort, and thus resolutely refused continued parenteral nutritional support. During hospitalization, we provided weekly cognitive-behavioral therapy and daily motivational interviewing to address her depression. However, the patient refused to continue after 2 sessions, stating, “I have no future; I am about to die. There is no point in these treatments.” This refusal came from her unresolved workplace bullying trauma, which left her feeling worthless and fatalistic, making it hard to engage with the therapy. The patient displayed pronounced pessimism throughout hospitalization, and this unaddressed psychological issue not only caused resistance to psychotherapy but also hindered nutritional compliance, slowing her hematopoietic recovery.

The core distinctiveness and clinical significance of this case lie in 3 aspects. Firstly, the patient’s BMI was as low as 11.7 kg/m^2^ (severe malnutrition), concurrently presenting with pancytopenia (marked reduction in all 3 blood cell lines)—a relatively uncommon combination in cases of AN with refeeding syndrome (RFS). Furthermore, the exclusion of malignancy through 2 bone marrow biopsies over 4 years of pancytopenia suggests that the core hematopoietic disorder stems from nutritional deficiency and abnormal bone marrow microenvironment (e.g., bone marrow adipose tissue infiltration). This provides a basis for differential diagnosis in AN patients presenting with “unexplained pancytopenia.” Secondly, the patient refused parenteral nutrition due to fear, achieving blood phosphorus levels from 0.32 mmol/L (critical value) to 0.8 mmol/L through a pure enteral stepwise regimen starting at 15 kcal/kg/d, incrementing by 5 kcal/kg/d every 24 hours, combined with intravenous phosphorus supplementation (0.6 mmol/kg/d). “d” combined with intravenous phosphate supplementation (0.6 mmol/kg d) rapidly corrected serum phosphorus from 0.32 mmol/L (critical level) to 0.85 mmol/L (normal range). This validated the feasibility of pure enteral nutrition in RFS management, offering a safe alternative for patients intolerant or refusing parenteral nutrition. Thirdly, the patient’s BNP elevation to 189.57 pg/mL stemmed not from cardiac dysfunction but from functional compensation for energy deficiency. Psychological trauma led to inadequate protein intake (40 g daily) and delayed hematopoietic recovery (leukocyte count still 3.1 × 10^9^/L 1 month post-discharge). This further underscores the clinical necessity for multidisciplinary motivational management in patients with anorexia nervosa complicated by RFS, with particular emphasis on improving psychological compliance.

## 6. Conclusion

In summary, hematological abnormalities frequently occur in patients with prolonged malnutrition, yet the emergence of refeeding syndrome is often overlooked in clinical management. The onset of refeeding syndrome may further exacerbate hematological damage through mechanisms such as hypophosphatemia. During prolonged malnutrition, persistent genetic alterations occur in the myeloid hematopoietic system, suggesting that the recovery of myeloid cell numbers and function post-refeeding may be limited. The superimposition of multiple clinical factors (such as persistent nutritional substrate deficiency, metabolic disturbances induced by refeeding syndrome, and chronic inflammatory states) may contribute to suboptimal therapeutic outcomes in promoting hematopoietic and immune function recovery. Although the occurrence of refeeding syndrome in anorexia nervosa patients is more likely related to the patient’s own nutritional status, high-calorie dietary regimens demonstrate greater advantages than traditional approaches in restoring patient weight and more effectively improving physical condition. This patient initially responded poorly to nutritional therapy; however, electrolyte levels gradually normalized following the introduction of enteral nutrition. This suggests that replacing conventional low-calorie feeding with high-calorie feeding, coupled with active correction of endocrine and hematopoietic microenvironment abnormalities, may yield superior therapeutic outcomes compared to traditional nutritional support regimens. Concurrently, such patients frequently present with multiple complications requiring multidisciplinary motivational management. Collaboration between nutrition, psychiatry, and general practice departments enhances diagnostic and therapeutic efficiency, builds patient trust, and prevents compliance issues from impeding treatment efficacy.

## Acknowledgments

We acknowledge the support provided by the medical staff of the Hematology Department at the Affiliated Hospital of Shandong Second Medical University in clinical data collection and case management.

## Author contributions

**Data curation:** Chunxiang Wang.

**Project administration:** Yuan Xue.

**Writing – review & editing:** Ying Guo.

**Writing – original draft:** Xin Zou.
